# S-Phase Kinase-associated Protein-2 Rejuvenates Senescent Endothelial Progenitor Cells and Induces Angiogenesis *in Vivo*

**DOI:** 10.1038/s41598-020-63716-y

**Published:** 2020-04-20

**Authors:** Hsueh-Hsiao Wang, Yi-Nan Lee, Cheng-Huang Su, Kuo-Tung Shu, Wen-Ting Liu, Chin-Ling Hsieh, Hung-I Yeh, Yih-Jer Wu

**Affiliations:** 1 Department of Medicine, MacKay Medical College, New Taipei City, 25245 Taiwan; 2 Cardiovascular Center, Department of Internal Medicine, and Departments of Medical Research, MacKay Memorial Hospital, Taipei, 10449 Taiwan

**Keywords:** Stem-cell biotechnology, Senescence

## Abstract

Cell cycle slowdown or arrest is a prominent feature of cellular senescence. S-phase kinase-associated protein-2 (Skp2), an F-box subunit of SCF^Skp2^ ubiquitin ligase, is a key regulator of G1/S transition. We investigated whether Skp2 plays a role in the regulation of endothelial progenitor cell (EPC) senescence, which is closely associated with aging-related vasculopathy. Replication-induced senescent EPCs demonstrated more pronounced senescence markers and lower Skp2 levels in comparison with those of their younger counterparts. Depletion of Skp2 induced increases in senescence-associated β-galactosidase (SA-βGal) activity and a reduction of telomere length and generated a senescent bioenergetics profile, whereas adenoviral-mediated Skp2 expression reversed the relevant senescence. EPCs isolated from older rats displayed a reduced proliferation rate and increased SA-βGal activity, both of which were significantly reversed by Skp2 ectopic expression. In addition to reversing senescence, Skp2 also rescued the angiogenic activity of senescent EPCs in the ischemic hind limbs of nude mice. The results revealed that ectopic expression of Skp2 has the potential to rejuvenate senescent EPCs and rescue their angiogenic activity and thus may be pivotal in the development of novel strategies to manage aging-related vascular disease.

## Introduction

Endothelial integrity is paramount for the maintenance of normal vascular function. Several cardiovascular (CV) risk factors, including smoking habit, diabetes, hypertension, and hyperlipidemia, have been shown to harm this integrity and result in endothelial dysfunction or even death. Without appropriate repair, blood vessels progress to advanced atherosclerosis, causing CV events to occur. The discovery of adult endothelial progenitor cells (EPCs) in 1997 greatly advanced knowledge of vascular repair^1^. A growing amount of evidence suggests that EPC number and function are reduced in patients with coronary artery disease or CV risk factors and that the number of circulating EPCs is positively associated with vascular function^2–4^. EPCs play a crucial role in the regulation of angiogenesis, which is vital to prevent organs or tissues from critical ischemia in terminal atherosclerotic diseases, in addition to vascular repair^3,5^.

Advanced age is a strong and independent risk factor for the development of atherosclerosis-related diseases. This is in line with evidence suggesting that the number of EPCs decreases with age in both humans and animals^6–8^. EPC senescence may account for this reduction and contribute to the occurrence of atherosclerosis in elderly patients. The therapeutic potential of EPCs has attracted considerable attention in the treatment of patients with ischemic cardiovascular disease or diabetic ischemic foot. However, the quantity and quality of EPCs limit autologous application in this population. Therefore, overcoming replication-induced senescence of EPCs to gain sufficient and functional cells would benefit older adult patients with diabetic ischemic diseases.

Cell cycle retardation, or G1 arrest, is a prominent feature of senescent cells^9^. Skp2 is an F-box protein of the Skp1/Cul-1/F-box (SCF) complex whose function is to reconstitute the E3 ligase for ubiquitination. In cell cycle regulation, Skp2 binds to critical cell cycle proteins such as p27^Kip1^ and targets them for ubiquitination and degradation, thus promoting cell proliferation. Studies have revealed that the PI3K/Akt pathway regulates the stability and activity of Skp2 in pro-oncogenic conditions. Akt interacts with and directly phosphorylates Skp2, triggering SCF complex formation and E3 ligase activity^10,11^, which in turn targets cell cycle proteins for degradation. The phosphorylation of Skp2 determines its stability and is mediated by phosphatase and tensin homolog (PTEN) in a PI3K/Akt pathway–dependent manner^12^. PTEN depletion causes a decrease in p27^Kip1^ levels with concomitant increase in Skp2, whereas ectopic PTEN expression leads to p27^Kip1^ accumulation, which is accompanied by a decrease of Skp2 in human glioblastoma cells^12^.

Skp2 is a crucial factor involved in cell cycle G1-S transition in vascular smooth muscle cells both *in vitro* and *in vivo*^13–15^. Skp2 is known for oncogene and therapy-induced senescence in tumor cell lines^16–18^, whereas the role of Skp2 in EPC senescence remains unclear. In the present study, we investigated the effects of replication-induced senescence on Skp2 expression in human EPCs. Senescent EPCs induced by serial passages exhibited lower Skp2 levels with more pronounced senescence markers than did those of their younger counterparts. To further confirm the crucial role of Skp2 in EPC senescence, Skp2 was depleted by siRNA in young EPCs. We observed that Skp2 silencing alone was sufficient to induce SA-βGal activity, reduction of telomere length, and a senescent bioenergetics profile. Notably, the ectopic expression of Skp2 in older EPCs was able to significantly suppress senescence markers and promote cell proliferation. EPCs isolated from 24-month-old rats exhibited profoundly reduced cell proliferation rate and increased SA-βGal activity, both of which were also significantly reversed by Skp2 overexpression. In addition to rejuvenating EPC senescence phenotypes, angiogenic activity was evaluated by injecting senescent EPCs with adenoviral-mediated Skp2 infection into the ischemic hind limbs of nude mice. The data revealed that Skp2 has potential to rejuvenate senescent EPCs and hence may be useful in the development of novel strategies to manage aging-related vascular disease.

## Experimental Procedures

### Human EPC isolation and senescence induction by repeated replication

This study was approved by the Mackay Memorial Hospital Institutional Review Board (ethical approval No. 13MMHIS114). All participants provided written informed consent, and all procedures adhered to the ethically approved protocol. Peripheral blood (80 mL) was collected from healthy donors. Peripheral blood mononuclear cells (PBMCs) were isolated from other blood components through centrifugation on Ficoll-Paque^TM^ plus (GE Healthcare, TX, USA) according to the manufacturer’s instructions. CD34^+^ cells were further isolated from PBMCs by using a CD34 MicroBead kit and MACSTM Cell Separation System (both from Miltenyi Biotec, Bergisch Gladbach, Germany) and then maintained in an endothelial cell growth medium (MV2 kit, PromoCell, Heidelberg, Germany) consisting of endothelial basal medium, 5% fetal bovine serum, human epidermal growth factor, vascular endothelial growth factor (VEGF), human fibroblast growth factor-B, insulin-like growth factor-1, ascorbic acid, and hydrocortisone. Cells (1 × 10^6^ cells/cm^2^) were seeded on fibronectin-coated dishes or cover slips (both from BD Biosciences, Bedford, USA) supplemented with endothelial cell MV2 medium and incubated in a 5% CO_2_ incubator at 37 °C. The medium was evaluated daily, and the first medium change was performed 2 days after plating. Subsequently, the culture medium was renewed every other day. A cobble-stone–like late-EPC colony could be observed after 2 weeks of culture. The colony was trypsinized with 0.05% trypsin–ethylenediaminetetraacetic acid (EDTA; Invitrogen, NY, USA) for passage. Late EPCs obtained through this protocol were further characterized and confirmed according to their ability to uptake 1,1′-dioctadecyl-3,3,3′,3′-tetramethyl-indocarbocyanine perchlorateacetylated low-density lipoprotein and bind *Ulex europaeus* agglutinin-1 lectin and their expression of VEGF, kinase insert domain receptor (KDR), and endothelial nitric oxide synthase (eNOS).

To obtain young and old EPCs, cells were grown in a medium and serially passaged until they reached passages 7–8 (young EPCs) or passages with cell doubling times (CDTs) that were twice as long (old EPCs) as those of the corresponding clones of young EPCs. To calculate CDT, 1 × 10^4^ cells were seeded on a 24-well plate in parallel with each passage and cultured for 48 hours. CDT was obtained using the following equation:

CDT = 48 × I/(F − I)

where F = final cell number and I = initial cell number, which was equal to 1 × 10^4^ in our setting.

### Cell cycle analysis

(1 × 10^6^) were fixed with ice-cold 70% ethanol before incubation with a propidium iodide (PI) solution (50 μg/mL PI, 0.1 mg/mL RNase A, 0.05% Triton X-100) at 37 °C for 40 min and then resuspended in 500 μL of phosphate buffered saline (PBS) for flow cytometry analysis using a FACScan flow cytometer (BD Biosciences).

### Senescence-associated β-galactosidase activity determination

Senescence-associated β-galactosidase (SA-βgal) activity was detected using a β-galactosidase staining kit (BioVision, Palo Alto, CA, USA) according to the manufacturer’s instructions. In brief, cells (1 × 10^4^) were washed in PBS and then fixed for 10–15 min at room temperature with 0.5 mL of a fixative solution. After being washed, the cells were incubated overnight with the staining solution at 37 °C. Cells were observed under a microscope at a magnification of 200× to monitor the development of blue color.

### Relative telomere length determination

Genomic DNA was extracted from the EPCs (1 × 10^5^) by using a High Pure PCR Template Preparation Kit (Roche, USA). Telomere length was estimated using a quantitative real-time polymerase chain reaction (Q-PCR). The telomere reaction mixture consisted of 1× SYBR Green Master Mix (Roche, USA), 270 nM telomere sense (GGTTTTTGAGGGTGAGGGTGAGGGTGAGGGTGAGGGT), and 900 nM telomere antisense (TCCCGACTATCCCTATCCCTATCCCTATCCCTATCCCTA). The reaction proceeded for 1 cycle at 95 °C for 10 min, followed by 25 cycles at 95 °C for 15 sec, 54 °C for 2 min, and 72 °C for 5.5 min. The 36B4 reaction (encoding acidic ribosomal phosphoprotein P0, serving as a single copy gene) consisted of 1x SYBR Green Master Mix, 300 nM 36B4 antisense (CAGCAAGTGGGAAGGTGTAATCC), and 500 nM 36B4 sense (CCCATTCTATCATCAACGGGTACAA). The 36B4 reaction proceeded for 1 cycle at 95 °C for 10 min, followed by 30 cycles at 95 °C for 15 sec, at 58 °C for 1 min, and 72 °C for 5.5 min. All Q-PCRs were performed in an ABI One Step Real-Time PCR machine (Applied Biosystems, Foster City, CA, USA). Relative telomere length (normalized T/S ratio) was calculated using the comparative Ct method after verification that the telomere and 36B4 Q-PCRs had equivalent amplification efficiencies.

### Cell growth and proliferation assay

Cell proliferation was evaluated through nuclear bromodeoxyuridine (BrdU) incorporation by using a BrdU immunochemistry kit (Millipore, USA). In brief, EPCs (1.5 × 10^4^/well) were seeded on coverslips in a 24-well plate and incubated with 10 μM BrdU for the final 8 hours of treatment. The cells were fixed with ice-cold 70% ethanol at 4 °C for 30 min after being washed. The BrdU-labeled cells were finally visualized using 3,3′-diaminobenzidine tetrahydrochloride staining according to the manufacturer’s instructions.

### Mitochondrial function evaluated according to oxygen consumption and extracellular acidification rates

Mitochondrial function was evaluated using a Seahorse Bioscience XF24 Extracellular Flux Analyzer (Bucher Biotec AG, Basel, Switzerland). EPCs (4 × 10^4^ cells/well) were seeded onto Seahorse Bioscience V7 tissue culture plates and allowed to grow for 24 hours before the measurement of oxygen consumption rate (OCR, pmol/min). Cells were grown in endothelial cell MV2 medium with growth factor supplement for one day before the experiment. On the day of metabolic flux analysis, cells were incubated at 37 °C in a non-CO_2_ incubator for 1 h with the medium changed to Customer Formulation Endothelial Cell BM MV2 (without glucose and sodium bicarbonate, c-97139, PromoCell). Initially, baseline cellular OCR was measured. For parameters of mitochondrial respiration, mitochondrial inhibitors oligomycin (10 μM), protonophore carbonyl cyanide m-chlorophenyl hydrazine (CCCP [50 μM]), and antimycin A (5 μM) (all from Sigma, St. Louis, MO, USA) were sequentially injected to inhibit adenosine triphosphate (ATP) synthase, mitochondrial membrane potential, and the electron-transport chain, respectively. OCRs were automatically calculated and recorded in real time by using Seahorse XF-24 software. The progress curves and data were calculated to demonstrate the relative contributions of basal, nonmitochondrial, ATP-linked, proton leak–linked, and maximal OCRs as well as cell reserve capacity (Fig. S1).

### Measurement of cellular ATP production

Total cell lysates were extracted from EPCs (1 × 10^4^) by adding 9 volumes of boiling 100 mM Tris (pH 7.7) and a 4 mM EDTA solution. The total lysates were quantified and subjected to ATP assay (ATP Bioluminescence Assay Kit CLS II, Roche) in accordance with the manufacturer’s instructions.

### Gene silencing mediated by siRNA

The siRNAs targeting human Skp2 and corresponding nonsense sequence used in the present study were designed and purchased from Invitrogen. Cells were transfected with 25 or 40 nM siRNA by using Lipofectamine RNAi Max Reagent (Invitrogen) in accordance with the manufacturer’s instructions. The cells were subjected to related analyses 48 hours after transfection.

### Adenovirus-mediated gene expression

Original stocks of recombinant adenoviral vectors encoding human wild-type Skp2 (Ad:WT-Skp2), luciferase (Ad:Luc), or β-galactosidase (Ad:βGal), driven by cytomegalovirus promoter, were generously provided by Dr. Mark Bond and Dr. Graciela B. Sala-Newby, Bristol Heart Institute, University of Bristol, United Kingdom. Recombinant adenoviruses were amplified in HEK 293 cells, purified by centrifugation through CsCl gradient, and titrated through plaque assay. Cells were infected with adenoviral vectors at a multiplicity of infection (MOI) of 100 for 3 hours to express genes of interest and subjected to related experiments 48 hours after infection.

### Western blotting analysis

Total cell lysates were prepared using an SDS-lysis buffer. Equal amounts of reduced proteins (20–50 μg) were separated through polyacrylamide gel electrophoresis and transferred onto a polyvinylidene fluoride membrane (Bio-Rad). Blots were blocked with 5% nonfat milk and then incubated with primary antibodies for the detection of senescence, cell cycles, or endothelial cell function proteins such as Sirt1 (Sigma), cellular senescence–inhibited gene (CSIG; Abcam), Skp2 (Cell Signaling), p21^Cip1^ (BD), p27^Kip1^ (BD), p16^INK4a^ (BD), p53 (Epitomic), Fis1 (Sigma), eNOS (BD), and plasminogen activator inhibitor-1 (PAI-1, Abcam). Appropriate secondary antibodies conjugated with alkaline phosphatase were used, and the chemiluminescence reaction was conducted using a VisiGlo substrate (Amresco) in accordance with the manufacturer’s instructions. Densitometry was performed by scanning the blots, which were then analyzed using TotalLab software (Nonlinear Dynamics).

### Animal experiments

The housing and care of animals and all procedures in this study adhered to the guidelines and regulations of the Republic of China Animal Protection Law (Scientific Application of Animals, 1998), and protocols were approved by the Animal Experiment Committee of Mackay Medical College, Taiwan (Approval No: A990007).

### Adenoviral expression of Skp2 in rat bone marrow mononuclear cells

Young (3-month old) and old (24-month old) Spraque–Dawley rats were killed to isolate PBMC and bone marrow mononuclear cells (BMMC), as described in the section of human PBMC fractionation. Both PBMC and BMMC were subjected to flow cytometry analysis to determine the percentages of CD34^+^ and KDR^+^ cells through immunocytochemical labeling with the specific antibodies phycoerythrin-conjugated mouse anti-CD34 monoclonal antibody (BD Pharmingen) and fluorescein isothiocyanate–conjugated rabbit anti-KDR monoclonal antibody (Cell Signaling). The remaining BMMC were maintained in an MV2 endothelial cell growth medium as described in the previous section. To test whether the adenoviral expression of Skp2 can rejuvenate BMMC isolated from old rats, BMMC were infected with adenoviral vectors expressing Skp2 or luciferase and then subjected to analysis to evaluate SA-βGal activity, cell proliferation, and the bioenergetics profile.

### Hind limb ischemia in nude mice and cell therapy

Female BALB/c athymic nude mice (8-weeks old, weighing 18–22 g) were anesthetized by intraperitoneal injection of pentobarbital (80 mg/kg). The right femoral artery and vein were ligated and severed from just above the deep femoral arteries to the popliteal artery and vein to create ischemic hind limbs. The right thighs and calves of animals were injected with one of the following reagents 24 hours after surgery: 1) 50 µL of PBS alone (PBS group); 2) PBS containing young EPCs (2 × 10^5^) (Y-EPC group); 3) PBS containing young EPCs (2 × 10^5^) infected with Ad:βGal at an MOI of 100 for 24 hours (Gal-Y-EPC group); 4) PBS containing old EPCs (2 × 10^5^) infected with Ad: βGal at an MOI of 100 for 24 hours (Gal-O-EPC group); and 5) PBS containing old EPCs (2 × 10^5^ cells) infected with Ad:Skp2 at an MOI of 100 for 24 hours (Skp2-O-EPC group). After 21 days, animals were killed by an overdose of intraperitoneal pentobarbital (100 mg/kg), and their calf muscles were dissected and processed for immunohistochemical analysis.

### Laser Doppler perfusion imaging

Hind limb subcutaneous perfusion was evaluated using a laser Doppler imager (Moor Instruments, Milwey, United Kingdom). Mice without amputated hind limbs were anesthetized through intraperitoneal injection of pentobarbital (80 mg/kg) and placed on a heater at 37 °C for 10 min before the ischemic legs and feet were scanned. Laser Doppler imaging was performed at 24 hours after surgery (just before the injection of EPCs) and then repeated on days 7, 14, and 21 after EPC injection, with perfusion status expressed as a ratio of right (ischemic) to left (normal) hind limb.

### Immunohistochemistry

Calf muscles were placed in 30% sucrose PBS for 24 hours, bisected at the middle level, mounted in an OCT compound (Leica), and snap-frozen in liquid nitrogen. To determine capillary density, calf muscles were mid-bisected with one cross-section 1.5 mm above the bisecting plane and another 1.5 mm below the bisecting plane; in other words, the two sections were 3 mm apart. Both sections, fixed with methanol for 10 min and washed briefly with PBS, were labeled with a mixture of monoclonal rat anti-murine platelet-endothelial cell adhesion molecule-1 antibody (1:200; B&D Pharmingen, San Diego, CA, USA) and polyclonal rabbit anti-laminin antibody (1:100; Chemicon) at 37 °C for 2 hours, followed by incubation with a mixture of CY3-conjugated anti-rat antibody and CY5-conjugated anti-rabbit antibody (both from Chemicon). In parallel, methanol-fixed frozen sections were stained with TRITC-conjugated murine EC-specific *Bandeiraea simplicifolia* lectin 1 (1:50; Sigma) and polyclonal rabbit anti-laminin antibody (1:100; Chemicon) at 4 °C overnight followed by incubation with a CY5-conjugated anti-rabbit antibody. Capillaries were counted for every 30 randomly chosen high-power fields on the two sections of each animal (without an amputated hind limb). The results are expressed as capillaries per myocyte.

### Statistical analysis

Continuous data are expressed as the mean ± standard deviation and were compared through a *t* test or Mann–Whitney test with unpaired data, as appropriate. Categorical data are presented as a ratio and were compared using a chi-square test or Fisher’s exact test. Data violating the principle of normal distribution across ordered groups were compared using a nonparametric trend test (Wilcoxon rank-sum test). One-way analysis of variance was used for an unadjusted comparison of three or more groups with post hoc Bonferroni correction for multiple comparisons between groups. All P values were two-sided, and a value less than 0.05 was considered statistically significant.

## Results

### Skp2 gradually decreased during the process of replication-induced senescence

**Figure 1:**
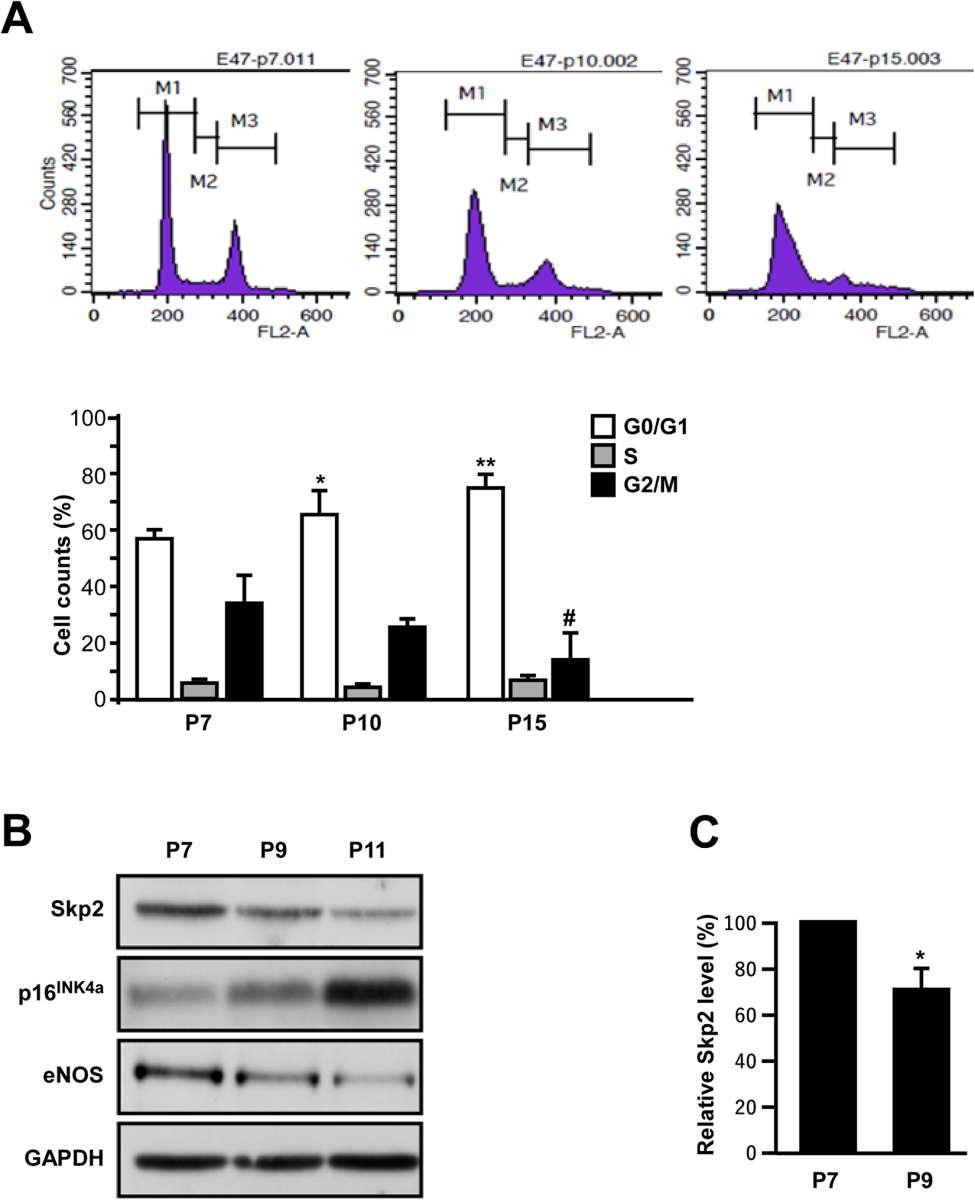
Effects of aging on EPC bioenergetics and of senescence on cell cycle transitions. (**A**) Replication-induced senescence increased the ratio of cells remaining in the G0 and G1 phase. A representative cell cycle analysis for different passages (P7, P10, and P15) of the same clone of EPCs revealed a graded increase in the percentages of G0 and G1 arrest along with the advance of the passages, whereas the percentages of cells in the G2 and M phase in the corresponding clone decreased with the increase in number of passages. Lower bar charts indicate the fractions of cell cycle transition among different passages of EPCs. The experiment was repeated with three different clones of EPCs with similar results. *Compared with G0 and G1, P < 0.05; **P < 0.01; ^#^compared with G2 and M, P < 0.05. (**B**) Skp2 expression was down-regulated along with the increased number of passages in the same EPC clone. Representative immunoblots of EPCs with serial passages presenting the expression of Skp2, senescence marker p16^INK4a^, and endothelial functional marker eNOS. The same results were obtained from three different clones of EPCs. (**C**) Quantification results of Skp2 level in young EPCs (p7) and EPCs with replication-induced senescence (p9) from three different clones. *Compared with p7, P < 0.05.

First, we assayed the effects of replication-induced senescence on the cell cycle transitions of EPCs. An increased percentage of EPCs remained in the G0 or G1 phase, and a decreased percentage of cells remained in the G2 or M phase after serial passages from P7 to P15 (Fig. 1A).

Because Skp2 is a major regulator of G1/S transition in many cell types, we investigated whether it plays a role in replication-induced EPC senescence. A comparison of EPC passages revealed that Skp2 levels gradually decreased with the increase of culture passages (Fig. 1B) and in three EPC clones (Fig. 1C). A consistent graded increase of senescence marker p16^INK4a^ was observed along with greater passage of EPCs, suggesting that serial replication induced EPC senescence. The stepwise decrease of eNOS in serial passages suggested that senescence impairs the angiogenic function of EPCs^19^.

### Depletion of Skp2 induces young EPC senescence and reduces mitochondrial respiratory efficiency

**Figure 2:**
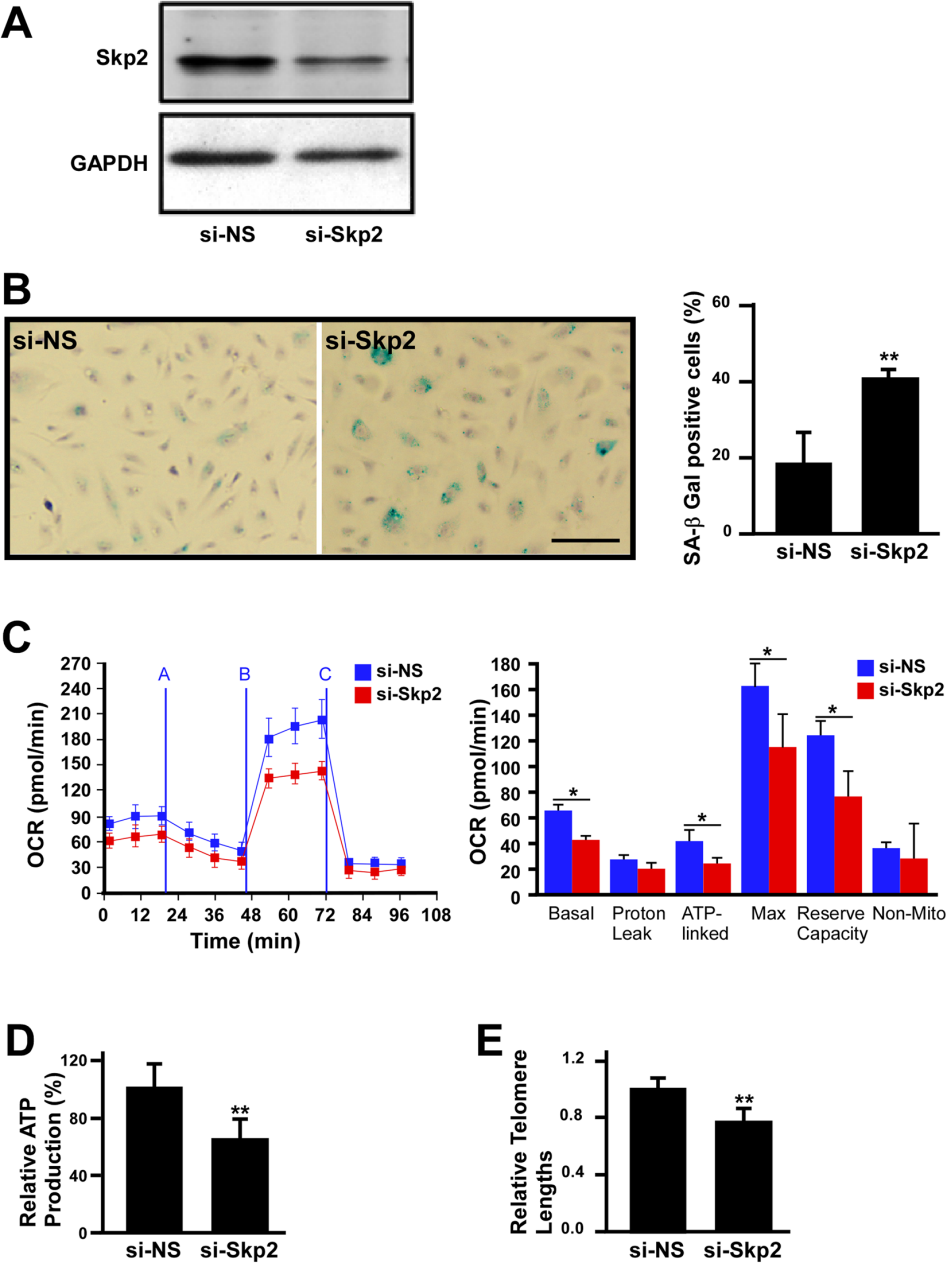
Depletion of Skp2 promotes EPC senescence and reduces respiratory efficiency. (**A**) Skp2 level in EPCs was reduced by siRNA treatment. Representative immunoblots of EPCs treated with nonsense (si-NS) or Skp2 siRNAs. Glyceraldehyde 3-phosphate dehydrogenase served as the loading control. Scale bar, 200 μm. (**B**) Depletion of Skp2 promoted EPC senescence. EPCs were treated with nonsense (si-NS) or si-Skp2 RNA and then subjected to SA-βGal staining (blue). Right panel: quantification of SA-βGal activity-positive cells for both groups. Data here were derived from three independent experiments. (**C**) Skp2 silencing altered the bioenergetics profiles of human EPCs. Left panel: representative OCR curves after the sequential addition of mitochondrial inhibitors in EPCs treated with si-NS or si-Skp2. Right panel: quantitative results for proton leak, cell reserve capacity, and basal, ATP-linked, maximal, and nonmitochondrial cell respiration. Data were obtained from four independent experiments. *Compared with si-NS, P < 0.05. Skp2 silencing also reduced ATP production (**D**) and EPC telomere length (**E**). Data here were obtained from three independent experiments, and relative ATP production and telomere length in si-NS–treated EPCs were set at 100% and 1.0, respectively. **Compared with si-NS, P < 0.01.

Because replication-induced senescence causes a reduction of Skp2 in EPCs, we tested whether the depletion of Skp2 is sufficient to induce EPC senescence. To this end, Skp2 was depleted by siRNA, which resulted in a pronounced decrease in protein level (Fig. 2A) and a significant increase in SA-βGal activity in young EPCs (Fig. 2B and quantification). We further assayed the effects of Skp2 depletion on the bioenergetics profile of EPCs. As revealed in in Fig. 2C, the OCR curve of Skp2 depletion moved downward compared with that of nonsense siRNA (si-NS) treatment, suggesting a decrease in respiratory efficiency. In parallel, Skp2 siRNA also reduced ATP production and telomere length (Fig. 2D,E). Accordingly, young EPCs with Skp2 depletion increased SA-βGal activity and reduced respiratory efficiency, ATP production, and telomere length to gain senescence phenotypes.

### Ectopic expression of Skp2 partially reverses EPC senescence

**Figure 3:**
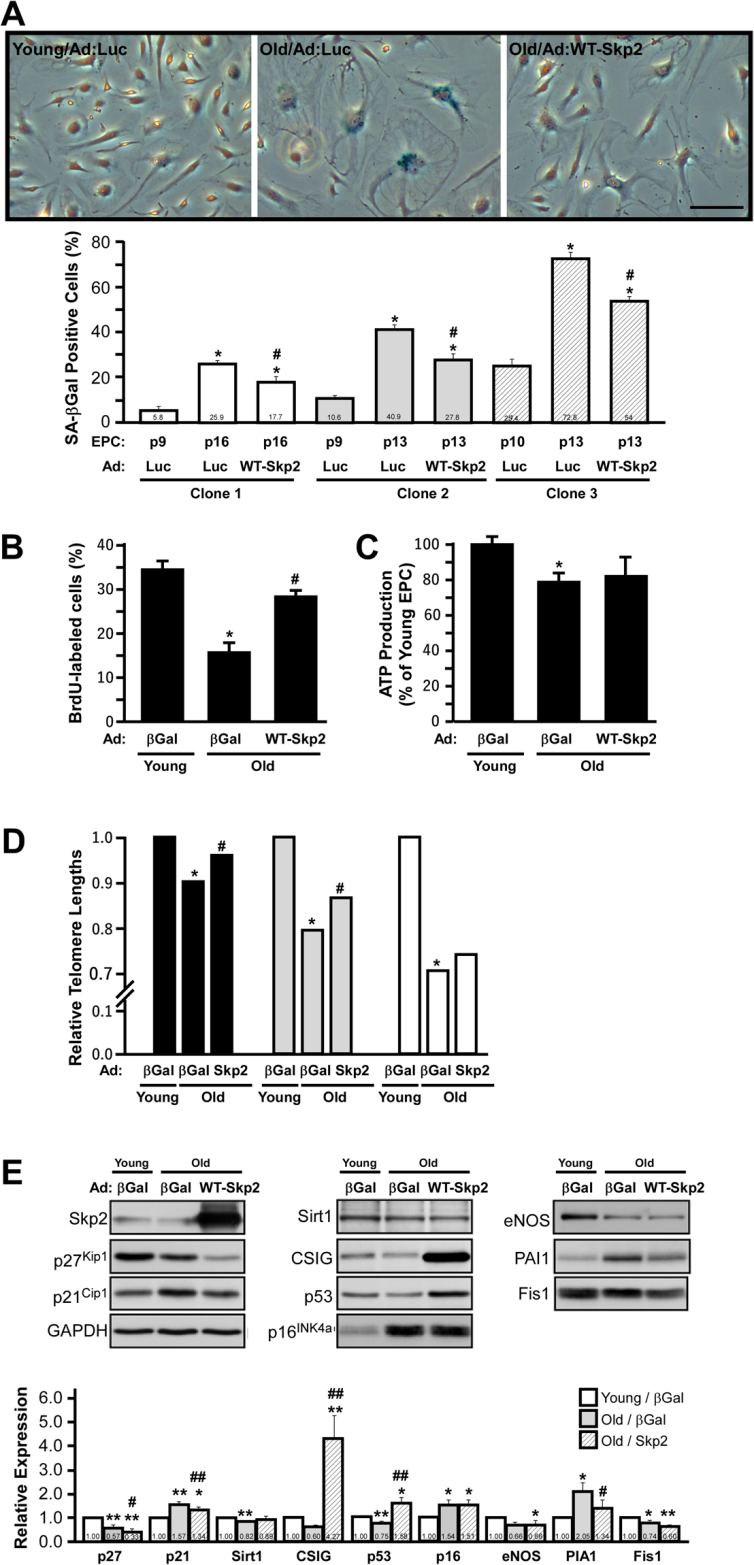
Ectopic expression of Skp2 partially reverses human EPC senescence. (**A**) Young and old EPCs were infected with adenovirus (Ad:) to express luciferase (Luc) or Skp2 (WT-Skp2). Cells were stained with X-Gal (blue) to determine SA-βGal activity. Lower panel: three individual EPC clones were compared with respect to the percentage of SA-βGal positive cells. *Compared with young/Ad:Luc, P < 0.05; ^#^compared with old/Ad:Luc, P < 0.05; scale bar, 100 μm. (**B,C**) ectopic expression of Skp2 partially rescued as a result of EPC proliferation and ATP production rates. (**D**) Telomere lengths were partially restored by adenovirus-mediated Skp2 (WT-Skp2) expression in three individual EPC clones. X-axis of (**B–D**) denote that EPCs (either young or old) are infected with adenovirus to express β-Gal (for DNA dosage compensation) or WT-Skp2 level. *Compared with young/Ad:Luc, P < 0.05; ^#^compared with old/Ad:Luc, P < 0.05. (**E**) Alteration of senescence-related protein levels according to Skp2 ectopic expression in young and old EPCs. Representative Western blots of young and old EPCs infected with adenovirus to express luciferase (Ad:Luc) or Skp2 (WT-Skp2) level. The expression of senescence-related proteins was detected by indicated antibodies. Glyceraldehyde 3-phosphate dehydrogenase served as the loading control. Lower panel: quantification of relative protein expressions detected using Western blots. Numbers indicate the relative densitometer reading comparing young EPCs (as control, 1.0). *Compared with young/Ad:Luc, P < 0.05; **P < 0.01. ^#^Compared with old/Ad:Luc, P < 0.05; #P < 0.01.

Next, we tested the gain-of-function effects of Skp2 on senescent EPCs. Young and old EPCs were infected with adenovirus to ectopically express luciferase (Ad:Luc) or Skp2 (WT-Skp2) in old EPCs. Ad:Luc did not increase the SA-βGal activity of young EPCs (data not shown), nor did it substantially affect the replication-induced increase of SA-βGal activity, which was, however, significantly dampened by Ad:WT-Skp2 infection (Fig. 3A). The effects of Skp2 on suppressing SA-βGal assay were consistently observed in three clones of EPCs harvested from different donors (Fig. 3A, quantification of the lower panel). The infection efficiency of adenovirus-mediated Sky2 expression was measured, and the results are displayed in Fig. 3E. In terms of proliferation and ATP production efficiency, the ectopic expression of Skp2 significantly rescued the reduced proliferation of old EPCs to an extent similar to that of young EPCs (Fig. 3B), but it did not appear to affect the reduced ATP production in old EPCs. Ad:WT-Skp2 only significantly rescued the telomere lengths in two of the three old EPC clones; however, it demonstrated the same trend in the third clone (Fig. 3D), with overall statistics indicating a significant trend for this rescue (paired *t* test, P < 0.05). Taken together, these results suggest that although ectopic expression of Skp2 alone is insufficient to comprehensively rejuvenate senescent EPCs, it does achieve this purpose at least partially (see the Discussion section for details).

### Effects of Skp2 ectopic expression on senescence-related proteins in old EPCs

Because the ectopic expression of Skp2 alone partially rejuvenates senescent EPCs, we surveyed a panel of senescence-related proteins affected by Skp2 overexpression in old EPCs (Fig. 3E). The protein levels of p21^Cip1^, p27^Kip1^, and PAI1 were markedly decreased, and CSIG and p53 expression were strongly induced by Skp2 expression (Fig. 3E). However, the effect of Skp2 on Sirt1, p16^INK4a^, eNOS, and Fis1 levels was unremarkable, suggesting that Skp2 alone may revert only some of the senescent mechanisms, or that Skp2 may alter these proteins independent of senescence (see the Discussion section for details).

### Skp2 rejuvenates old EPCs

**Figure 4:**
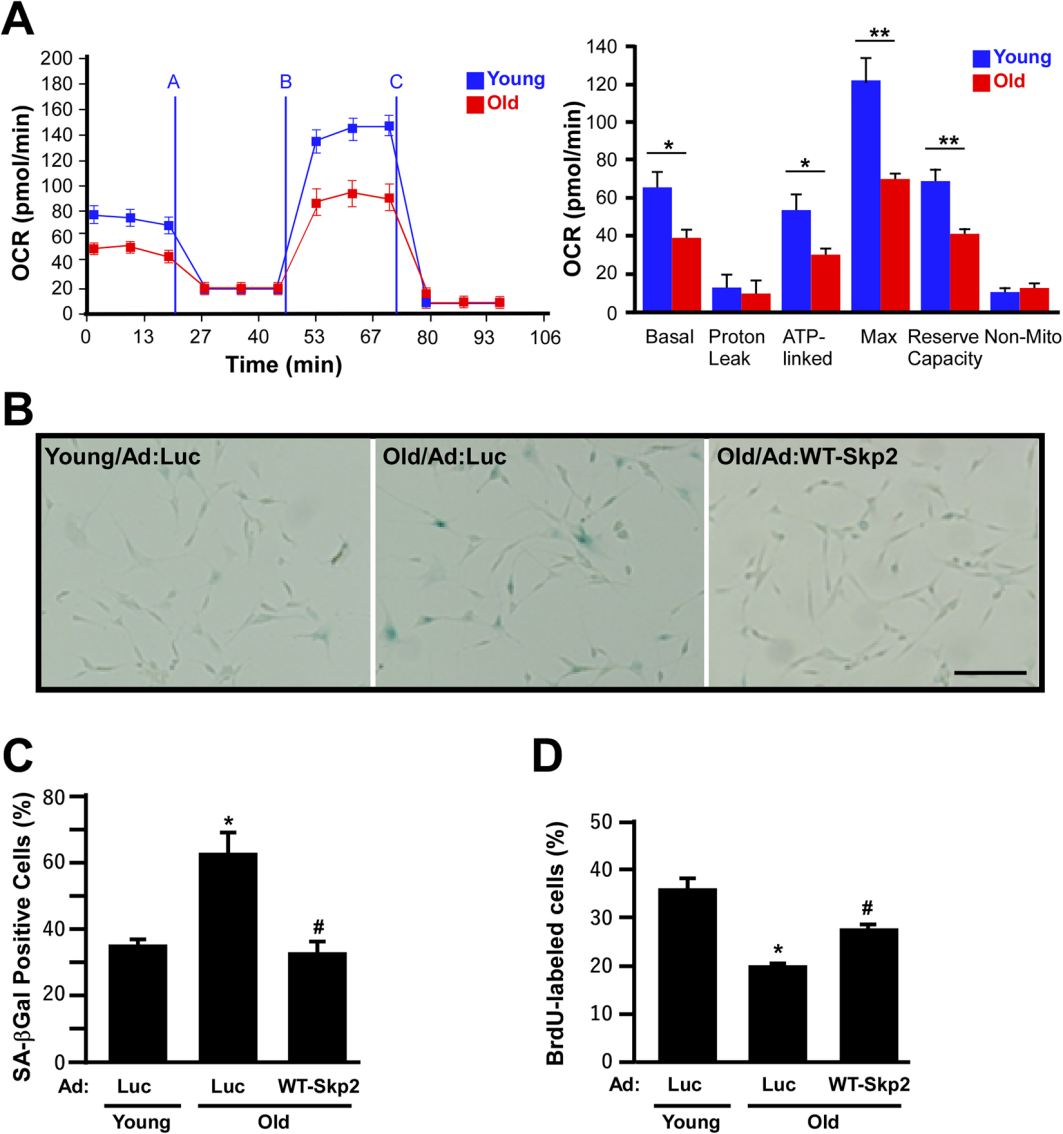
Skp2 rejuvenates old rat EPC senescence phenotypes and increases respiratory efficiency. (**A**) Bioenergetics profiles of EPCs isolated from young (3-month old) and old (24-month old) rats. EPCs (3 × 10^4^ cells/well) were seeded onto Seahorse Bioscience V7 tissue culture plates for 24 hours. Respiratory inhibitors were injected at the indicated time (**A**, oligomycin; **B**, CCCP; and **C**, antimycin A) to determine proton leak respiration, maximal respiratory capacity, and mitochondrial reserve capacity, respectively. Right panel: quantitative comparisons of OCRs between EPCs harvested from young and old rats. Despite proton leak and nonmitochondrial respiration, all of the OCR parameters in the old group were significantly decreased. *P < 0.05; **P < 0.001. (**B**) Rat EPCs were stained with X-Gal (blue) to determine SA-βGal activity. EPCs were harvested from young (3-month old) and old (24-month old) rats with luciferase (Luc) or Skp2 (WT-Skp2) infection. Scale bar, 100 μm. The percentages of X-Gal positive and BrdU-labeled cells are indicated in (**C**,**D**).

Because the depletion of Skp2 induces young EPCs to gain senescent phenotypes and reduces mitochondrial respiratory efficiency, we evaluated whether geriatric stress caused *in vivo* EPC bioenergetic profile changes. EPCs isolated from 3- and 24-month-old rats (young and old groups, respectively) were seeded onto Seahorse culture plates for OCR assay to compare their respiratory efficiency. In general, the OCR of the young group was greater than that of the old group (Fig. 4A). Comparison of individual parameters revealed that despite proton leak and nonmitochondrial respiration, all OCR parameters were significantly decreased in the old group, suggesting that geriatric stress reduced EPC mitochondrial respiration efficiency (Fig. 4A, right panel for quantification). Notably, this *in vivo* EPC senescence pattern was perfectly consistent with that in the *in vitro* Skp2-silenced EPCs (Fig. 2C).

Next, we tested whether the ectopic expression of Skp2 could rejuvenate old EPCs. EPCs isolated from young and old rats were infected with Ad:Luc (as the vehicle control) and Skp2 (WT-Skp2) to assay their senescence phenotypes (Fig. 4B,C). EPCs harvested from old (24-month old) rats exhibited approximately twice the amount of SA-βGal activity as that of EPCs harvested from young (3-month old) rats (Fig. 4B, Young/Ad:Luc vs. Old/Ad:Luc). The SA-βGal activity decreased to the level of EPCs isolated from young rats when old rat EPCs were infected with Skp2 (Old/Ad:WT-Skp2). In addition, Skp2 overexpression also appeared to rescue senescent cell morphology to some extent (“sunny-side-up fried egg” cells, see Fig. 4B). Consistently, the BrdU incorporation rate of old rat EPCs was also significantly increased by Skp2 ectopic expression, suggesting that Skp2 can phenotypically and functionally rejuvenate old EPCs (Fig. 4D).

### Skp2 restores the angiogenic ability of senescent human EPCs *in vivo*

**Figure 5:**
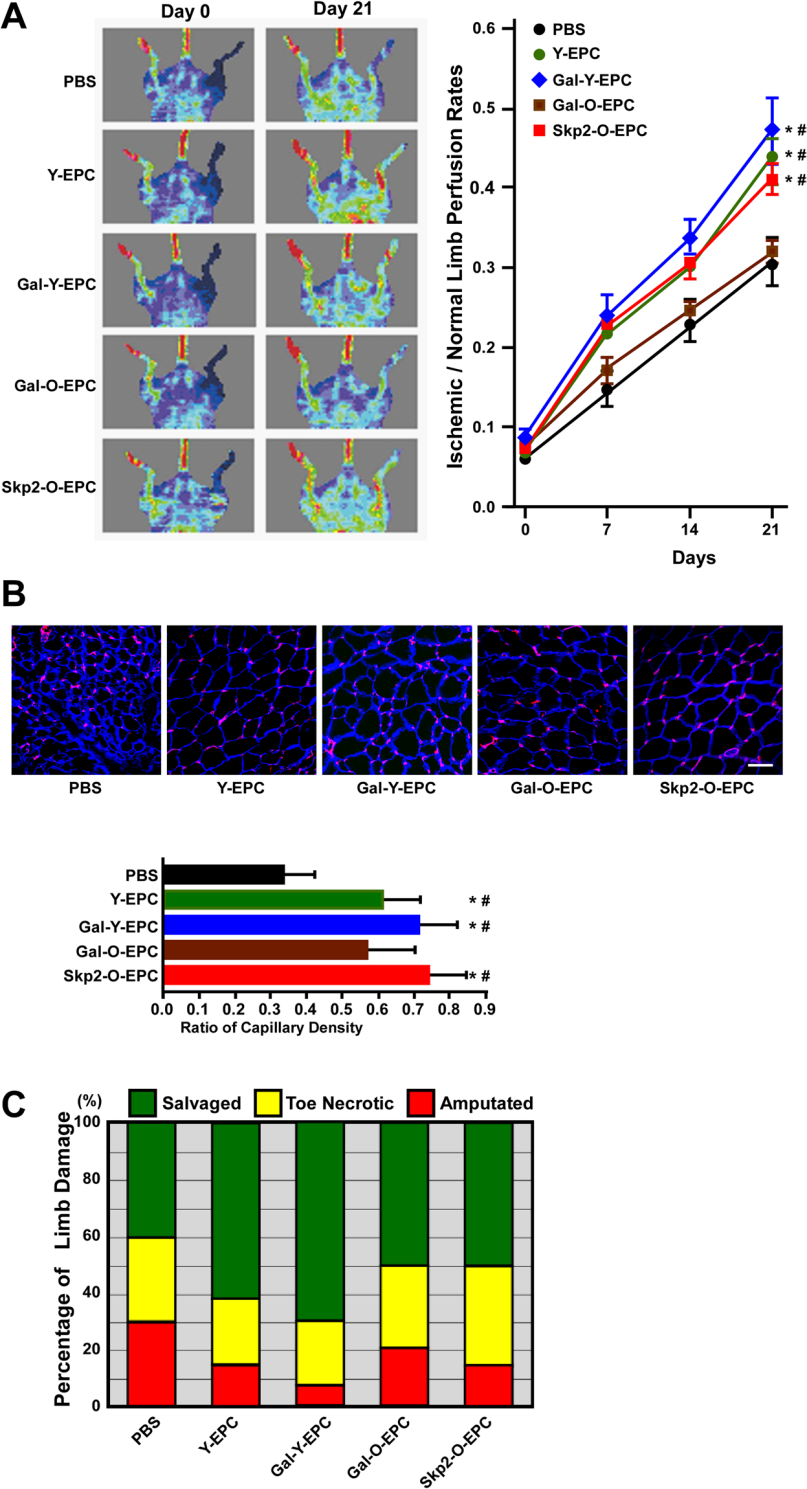
Skp2 restores the angiogenic activity of senescent human EPCs *in vivo*. (**A**) Laser Doppler perfusion images of hind limb ischemic rats injected with human EPCs to test their angiogenic activity. Young and old EPCs were infected with Gal4 (Gal) or Skp2 and imaged at day 0 and day 21 after EPC injection. PBS was the group without EPCs as mock injection. Right hind limbs were untreated to serve as the control. Right panel: quantification of the ischemia area versus the normal perfusion area. The days on which the images were captured are indicated. *P < 0.05 compared with the PBS group; ^#^P < 0.05 compared with the Gal-O-EPC group. (**B**) Representative images of myocyte and capillary staining. At day 21, tissues were sectioned and stained with laminin (blue) and lectin1 (red) to visualize myocytes and capillaries, respectively. Lower panel: quantification of capillary density, which was derived as the number of myocytes divided by the number of capillaries. Notably, because of tissue atrophy, the peripheries of PBS-treated myocytes appeared to be smaller than those of the EPC-injected myocytes. *P < 0.05 compared with the PBS group; #P < 0.05 compared with the Gal-O-EPC group. Scale bar, 50 μm. (**C**) Damaged tissue was rescued through the injection of young EPCs and Skp2-infected old EPCs. The degree of tissue damage was classified into three categories: salvageable (green), toe necrotic (yellow), and amputated (red) for each group (n = 10). Notably, the percentage of amputated limbs in the Skp2-treated old EPC group (Skp2-O-EPC) was lower than that in the Gal4-treated old EPC group (Gal-O-EPC).

Several studies have reported that senescence impairs EPC angiogenic activity, resulting in the failure of cell therapy. We tested whether the angiogenic ability of senescent human EPCs was restored *in vivo* by Skp2 ectopic expression. To this end, female athymic nude mice with ischemic hind limbs were injected with young or old human EPCs with adenovirus-mediated βGal (βGal served as the control group) or Skp2 expression (Fig. 5A). Hind limb subcutaneous perfusion was evaluated using a laser Doppler imager. Mice injected with old EPCs (Gal-O-EPC) exhibited a poor perfusion recovery curve, similar to that of the PBS injection group. When mice were injected with old EPCs with Skp2 ectopic expression (Skp2-O-EPC), their perfusion rate restored to a level close to that of the young EPC groups (Y-EPC and Gal-Y-EPC).

Next, we examined the capillary density of calf muscles isolated from ischemic hind limbs on day 21. Tissues were stained with laminin (blue) to delineate the basement membrane of myofiber and *Bandeiraea simplicifolia* lectin 1 (red) to detect murine endothelial cells (Fig. 5B). Ischemic tissues injected with young EPCs (Y-EPC or Gal-Y-EPC) exhibited a higher capillary density compared with that of the sham injection (PBS). The capillary density in ischemic hind limbs injected with Skp2-expressed old EPCs (Skp2-O-EPC) was higher than that in ischemic hind limbs injected with control adenovirus-infected old EPCs (Gal-O-EPC). Similar to the results for perfusion recovery, the capillary density of Gal-O-EPC-injected ischemic limbs was not statistically different from that in the PBS-injected limbs. Regarding ischemic limb conditions (Fig. 5C), the two young EPC groups demonstrated better recovery of ischemic limb than the other groups. Although Skp2-expressed old EPC injection appeared to have the same limb-salvaging effect as βGal-expressed old EPC injection, the Skp2-expressed injection tended to exhibit a better trend of avoiding severe limb conditions (amputation).

## Discussion

Aging, an independent risk factor for ischemic cardiovascular diseases, systemically affects organism activity and function. EPCs can play a powerful role in cell therapy for limb ischemia resulting from diabetes and aging. However, the number of “effective” EPCs is inversely related to increase in age^6,7^. We compared the bioenergetics profiles of EPCs from young and old rats, the results of which revealed a decrease in respiratory efficiency in older animals (Fig. 4). Notably, the introduction of Skp2 into senescent EPCs restored their angiogenic activity in rescuing the perfusion of ischemic tissues. To our knowledge, this is the first study to report using Skp2 expression to rejuvenate senescent EPCs and thus reveal the therapeutic potential of Skp2 for treating ischemic cardiovascular diseases.

One study reported that replication-induced senescence in fibroblasts causes a metabolism shift with an increase of glycolysis and decrease of ATP production, leading to proliferation arrest^5^. In the present study, we observed that series cell passages increased the EPC doubling time and percentage of cells remaining in the G0 and G1 phase, while the percentage of cells remaining in the G2 and M phase decreased (Fig. 1A). During the process of replication-induced senescence, the level of Skp2 decreased as the number of passages increased (Fig. 1B,C), suggesting the gradual Skp2 downregulation following a series of passages may contribute to cell senescence. One study reported that through genetic depletion, Skp2 played a direct role in cellular senescence, and this role was independent of tumor suppressors, such as PTEN, or the alternative reading frame protein (ARF)/p53 pathway^18^. Therefore, we evaluated whether Skp2 depletion alone is sufficient to induce EPC senescence. To this end, we performed a Skp2 loss-of-function test on young EPCs, which revealed decreased OCR, ATP production, and telomere length and increased senescence marker SA-βGal expression (Fig. 2B). Thus, the results suggest that Skp2, in addition to regulating G1/S transition, participates in the process of EPC senescence.

Studies have indicated that the number of EPCs in elderly people is decreased^6,7^. Older adult patients and those with diabetes are more susceptible to ischemic diseases. Therefore, after revealing that depletion of Skp2 leads to EPC senescence, more crucially, we were eager to investigate whether the introduction of Skp2 into senescent EPCs could rejuvenate phenotypes or restore their angiogenic activity. To this end, EPCs were harvested from three donors to evaluate whether EPCs from different individuals would reveal the same trend. We observed that the ectopic expression of Skp2 could reduce the percentage of SA-βGal positive cells in all EPCs from different donors, suggesting the potential of Skp2 to reverse senescence (Fig. 3A). The effects of replication-induced senescence on EPC proliferation and telomere length were also consistently reversed by the introduction of Skp2 expression into higher-passage EPCs (Fig. 3B,D).

We found that Skp2 expression failed to rescue the decrease of ATP production in senescent EPCs (Fig. 3C), which is in line with our observation (Fig. 3E) that Skp2 did not regulate EPC senescence-induced downregulation of mitochondrial fission protein, Fis1^20^. This fact that single-factor recovery by Skp2 is not sufficient to restore all senescent phenotypes, which are involved in intricate senescent mechanisms, is perfectly reasonable.

In our global survey of senescence-related proteins, CSIG was the most strongly induced by Skp2 ectopic expression in senescent EPCs, but Skp2 had no effect on the expression of p16^INK4a^ (Fig. 3E). This is consistent with the findings of a different study, which reported that the ectopic expression of CSIG did not influence p16^INK4a^ expression and that CSIG negatively regulated p27^Kip1^ expression to promote cell proliferation by inhibiting PTEN translation^21^. Skp2 may stabilize CSIG through nonproteolytic polyubiquitination, as demonstrated by its regulation of yes-associated protein and liver kinase B1^22,23^, and thereby promote p27^Kip1^ degradation. Although p53 expression was also induced by Skp2 ectopic expression, its level actually was decreased in replication-induced old EPCs (Fig. 3E). This result suggests that p53 may not be the primary regulator of replication-induced senescence in EPCs. Therefore, the increase of p53 level in the cell content of EPCs, induced by Skp2 overexpression (old/WT-Skp2), may not account for the induction of cellular senescence. Emerging evidence suggests that cellular senescence can be triggered in an ARF/p53-independent manner^17,18,24^. The final p21^Cip1^ levels are determined through the combined effect of Skp2 and p53, which in turn increase p21^Cip1^ degradation^25^ and transcription, respectively. The changes pattern of p21^Cip1^ observed in our stud implies that Skp2 action may play a more dominant role than p53 in EPCs. Although p27^Kip1^ is also a substrate for Skp2 (similar to p21^Cip1^), replication-induced Skp2 downregulation did not lead to a corresponding increase of p27^Kip1^ level, suggesting that p27^Kip1^ is regulated in the replication-induced senescence through transcription or other Skp2-independent pathways. Despite its failure to rescue Sirt1 and eNOS, ectopic Skp2 expression significantly limited the increase of PAI-1 level, which is a crucial maker for endothelial dysfunction. Although Fig. 3E indicated variable changes in cell cycle regulators along with senescence and post Skp2 ectopic expression, recent studies have revealed that cell cycle arrest can be uncoupled with senescent phenotypes^26,27^. Therefore, the effects of Skp2 overexpression on cell cycle regulator levels may not be relevant to senescence reversal.

To the best of our knowledge, this study is the first to examine the angiogenic activity of human EPCs rejuvenated by Skp2 ectopic expression *in vivo* (Fig. 5). Rejuvenated EPCs improved capillary growth and peripheral blood perfusion and rescued ischemic tissues. Our study demonstrated the proof-of-concept that the introduction of Skp2 into senescent EPCs can overwhelm cycle arrest and not only reverse senescent phenotypes but also restore angiogenic activity *in vivo*.

Previous reports have indicated that Skp2 proteins were increased in several cancer cell lines and acquired proto-oncogenic potential from the coexpression of constitutively active H-Ras^G12V^ oncogenes^19,28^. However, there are no reports to date claiming that Skp2 overexpression alone is sufficient to transform primary culture cells into malignant tumors. EPCs with Skp2 ectopic expression did not cause any abnormal angiogenesis, colony formation, or metastasis in our 21-day cell therapy for ischemic hind limbs. Indeed, Delogu, S. *et al*. reported that the forced overexpression of Skp2 alone did not induce tumor development^29^. Therefore, the term “oncogenic” should be used with caution when describing Skp2.

The current study revealed that the ectopic expression of Skp2 alone is sufficient to rejuvenate EPCs, which were able to rescue ischemic tissue damage in an animal study. These findings demonstrate that Skp2 has the potential to rejuvenate senescent EPC and, hence, may prove useful in the development of novel strategies to manage aging-related vascular disease.

## Supplementary information

Supplementary Figures
