# Supplementary material for: S-Phase Kinase-associated Protein-2 Rejuvenates Senescent Endothelial Progenitor Cells and Induces Angiogenesis in Vivo
Source: Sci Rep. 2020 Apr 20;10:6646. doi: 10.1038/s41598-020-63716-y (PMC7171137; doi:10.1038/s41598-020-63716-y)

S-phase kinase-associated protein-2 rejuvenates senescent endothelial progenitor cells and imposes angiogenesis *in vivo*

Hsueh-Hsiao Wang[2*], Yi-Nan Lee[1*], Cheng-Huang Su[1], Kuo-Tung Shu[1], Wen-Ting Liu[1], Chin-Ling Hsieh[1], Hung-I Yeh[1,2], Yih-Jer Wu[1,2⁋]
[1]Cardiovascular Division, Department of Internal Medicine, and Departments of Medical Research, Mackay Memorial Hospital, Taipei 10449, Taiwan.
[2]Department of Medicine, Mackay Medical College, New Taipei City 25245, Taiwan.
⁋ Address correspondence to: Dr Yih-Jer Wu, Department of Medicine, Mackay Medical College, No. 46, Section 3, Jhong-Jheng Road, San-Jhih, New Taipei 25245, Taiwan.

Fig. S1

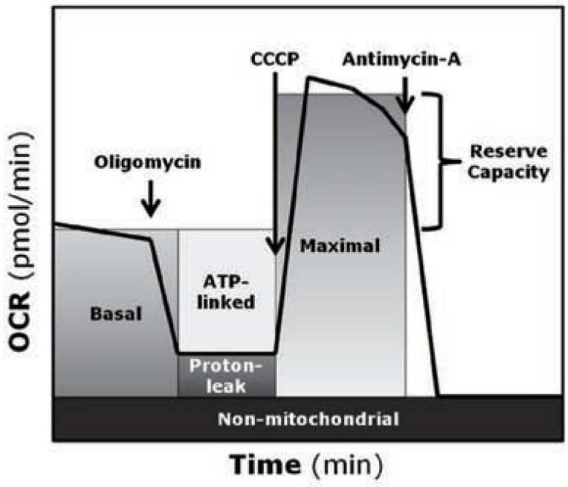

Bioenergetics profile of EPCs

EPCs were incubated at 37 ℃ in a non-CO_2 incubator for 1 h with Customer Formulation Endothelial Cell BM MV2 (without glucose and sodium bicarbonate, c-97139, PromoCell). Initially baseline cellular OCR was measured. For parameters of mitochomdria respiratory, mitochondrial inhibitors oligomycin (10 μM), protonophore carbonyl cyanide m-chlorophenyl hydrazine (CCCP (50 μM)), and antimycin-A (5 μM) (all from Sigma, St. Louis, MO, USA), were sequentially injected to inhibit ATP synthase, mitochondrial membrane potential, and electron-transport chain, respectively. OCRs were automatically calculated and recorded in real time by the Seahorse XF-24 software.

Fig. 1B

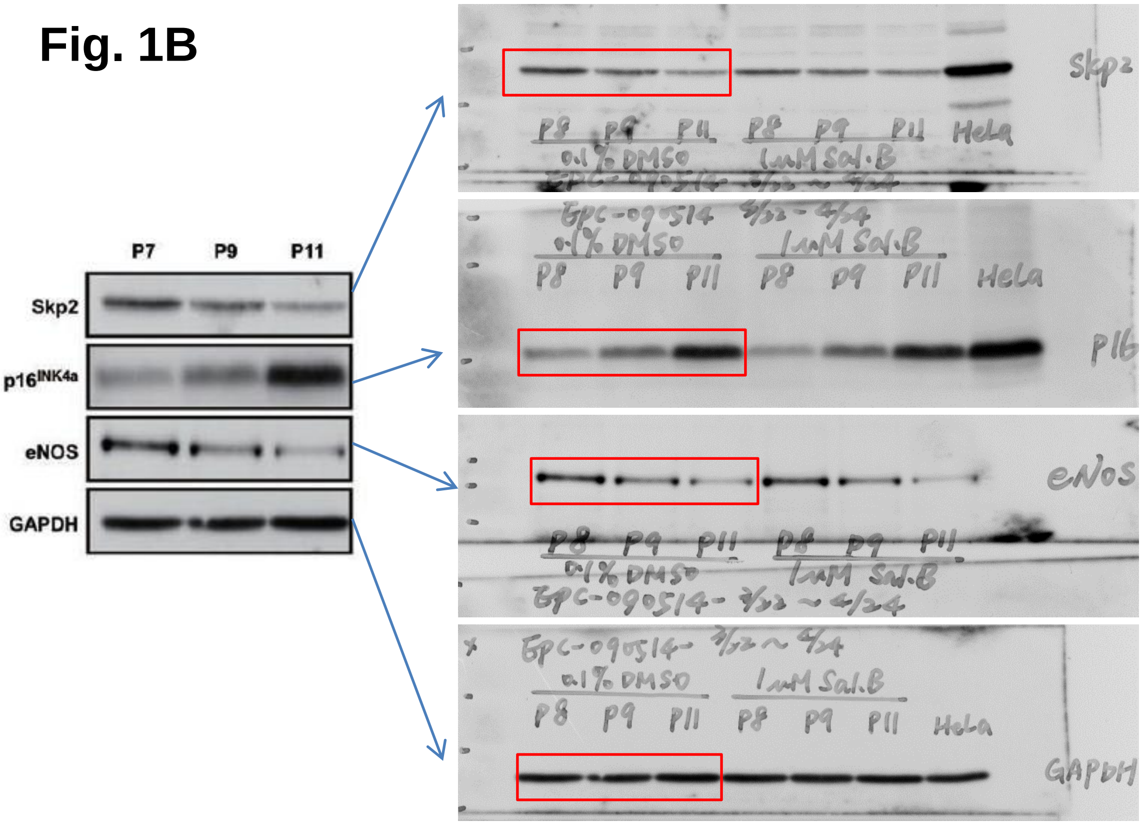

Fig. 2A

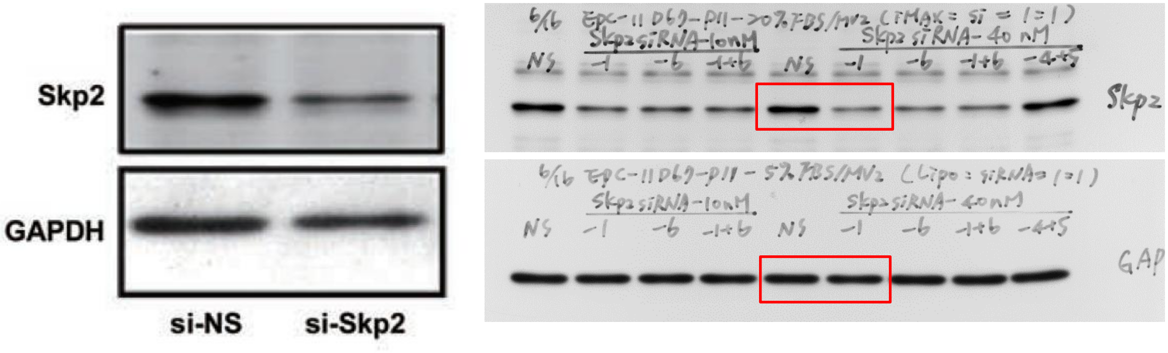

Fig. 3E

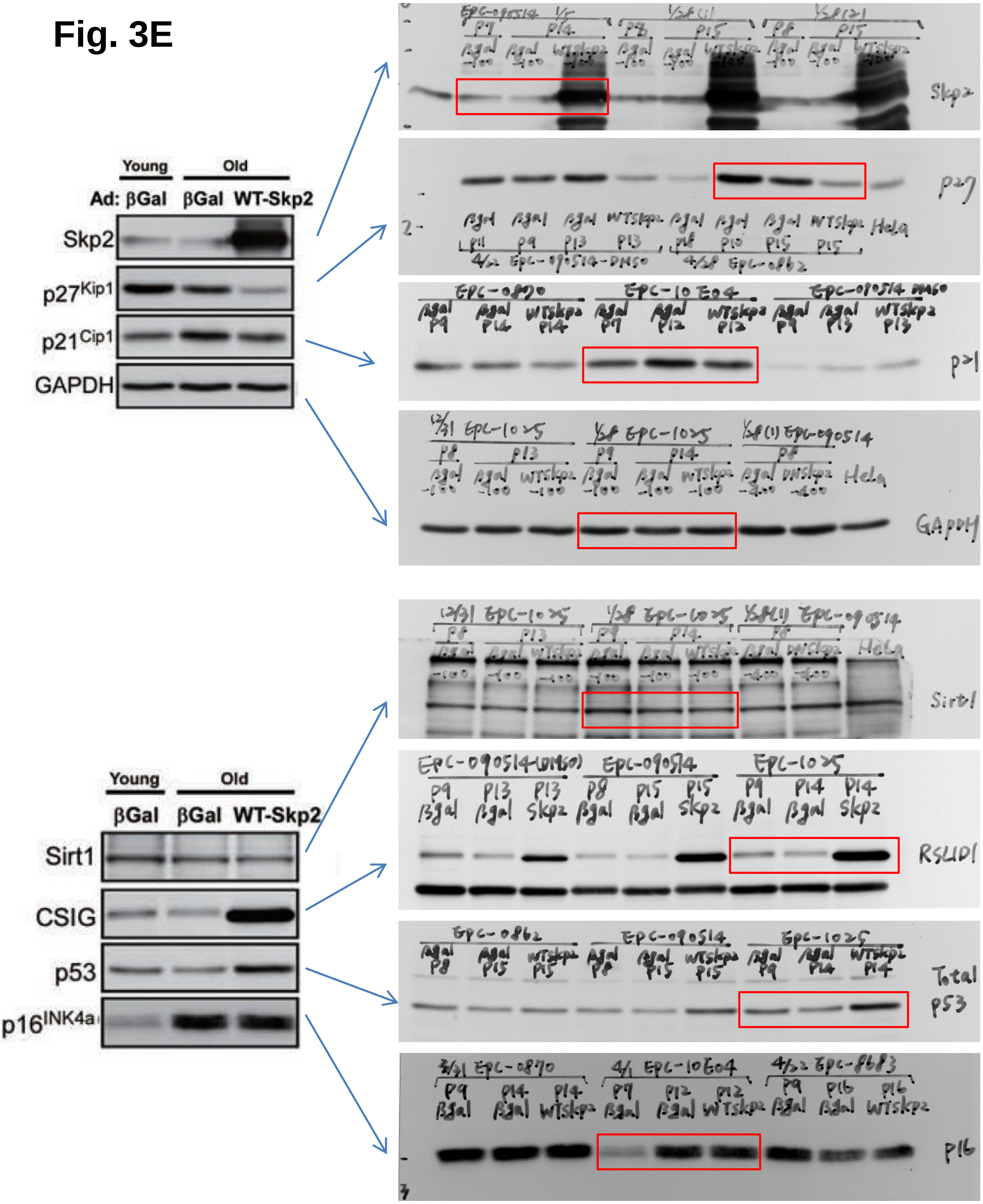
Young
Old
Ad: βGal
βGal
WT-Skp2
Skp2
p27Kip1
p21Cip1
GAPDH
Young
Old
βGal
βGal
WT-Skp2
Sirt1
CSIG
p53
p16INK4a

Fig. 3E (continue)

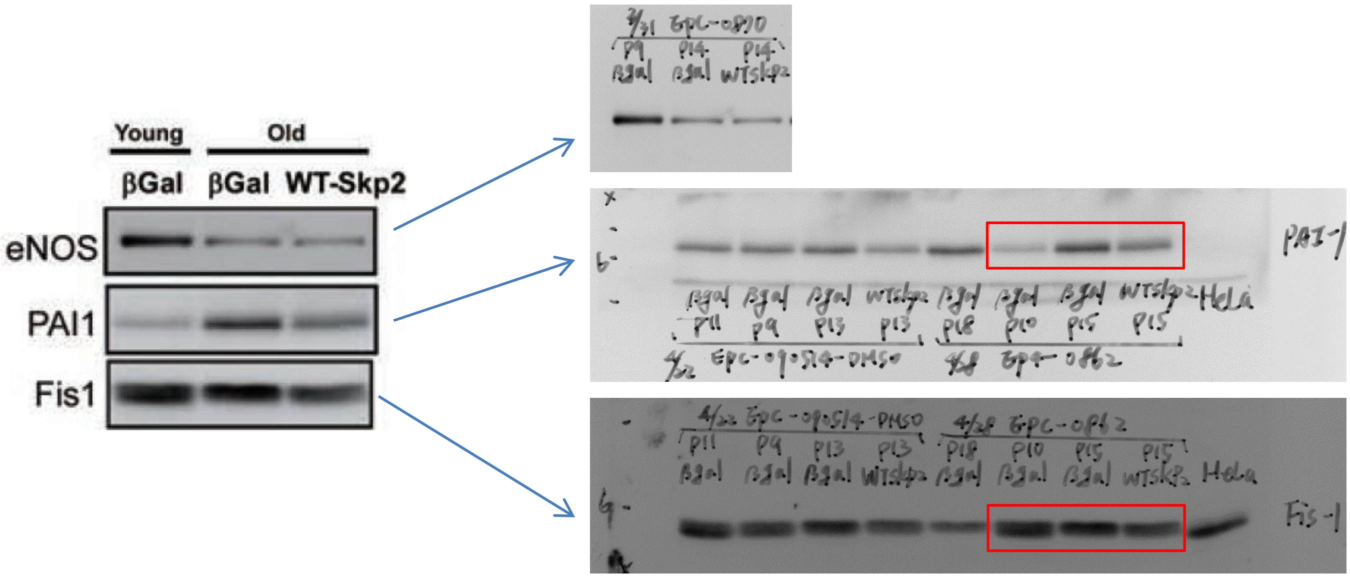

Supplement: Supplementary file 1 — Supplementary Figures [file 41598_2020_63716_MOESM1_ESM.pdf]
